# Editorial to the Special Issue—“Technology for Natural Products Research”

**DOI:** 10.3390/molecules25020327

**Published:** 2020-01-14

**Authors:** Brendan M. Duggan

**Affiliations:** Skaggs School of Pharmacy and Pharmaceutical Sciences, University of California, San Diego, La Jolla, CA 92093, USA; bmduggan@ucsd.edu

Natural product research continues to be a productive source of unusual chemistry, producing novel compounds for biomedical applications and, increasingly, sustainably providing commercially useful compounds. Despite its long history, the technologies used in natural products research are still amenable to improvements and the introduction of novel methods, materials and reagents. This special issue highlights several recent developments and improvements in the technology for natural products research.

In recent years, laboratory culture of microbes has expanded the ability of natural products researchers to examine a wider variety of organisms and facilitated the isolation and identification of rare compounds. Gurusinghe et al. [1] compared the use of standard media with a “rhizochip” device which provided enhanced sampling of the rhizosphere microbiota. They were able to recover organisms not found by other, more traditional methods. The laboratory culture of some of these organisms has led to the identification of several new and unusual metabolites.

Biological material for natural products research is often collected in remote areas with limited access to sample storage or processing technology. Collection and sample processing methods can thus impact what can be identified or isolated from the material in a laboratory setting. Juárez-Aragón et al. [2] quantified the impact of a variety of drying methods on the phenolic and flavonoid content and free radical scavenging potential of medicinal plants in the *Rhus* genus. They report that not only did the method used affect the metabolic content, but that the impact of the methods was species-dependent as well.

Solvent extraction is another step that greatly influences the outcome of a natural products program and for this reason alternatives to traditional organic solvents have been examined. The use of supercritical CO_2_ extraction to obtain commercially useful compounds from microalgae was evaluated by Molino et al. [3] and Mehariya et al. [4]. Both groups report the impact of mechanical pre-treatment methods and the optimal pressure, temperature and flow rates for maximal recovery and highest purity of the desired compounds. Extraction methods can also be tailored to target specific compounds. Yohannes et al. [5] used ionic liquids and ultrasonication to specifically extract alkaloids from the Chinese medicinal plant *Radix physochlainae*. Their protocol was quicker and more efficient than traditional methods.

In order for traditional medicines and herbal extracts to be safely introduced to the market, methods for verifying the provenance, composition and quality of the material are needed. Lee et al. [6] present a UPLC-QTOF/MS analysis of processed ginseng products. Principle component analysis of the results was able to differentiate the products. A similar approach was used by Yuan et al. [7] who coupled gas chromatography to QTOF/MS to study blueberry wines and, again, used principle component analysis to distinguish wines derived from different cultivars. Cabanas-Garcia et al. [8] used UPLC-MS/MS to profile the metabolome of a cactus used in folk medicine and suggested to produce compounds with psychotropic properties. They detected 69 compounds and were able to identify 60 of them by comparison with literature retention times, molecular masses and fragmentation patterns.

Tsang et al. [9] present an innovative approach to quality control of a traditional Chinese medicine, Dictamni cortex, used for the treatment of inflammatory diseases. Aqueous and ethanolic extracts of the herbal material applied to human peripheral blood mononuclear cells induced changes in the production of inflammatory chemokines and cytokines, which were monitored by flow cytometry. This high throughput method was able to distinguish material with different origins and quantify its immunomodulatory activity.

Biological activity can be modified dramatically by stereochemistry; thus, its determination is a critical component of natural product structure elucidation. Pearce et al. [10] present new, more robust methods for the determination of the stereochemistry of amino alcohols. Application of their methods to multiple collections of a compound with disputed configuration showed that the stereochemistry varied with the collection, suggested to be the result of “promiscuous biosynthesis”.

As the papers in this special issue show, many different facets of technology for natural product research continue to be explored. The processes impacting compound identification and discovery are being probed so that they may be better controlled, and the desired outcomes obtained. Methods to enhance quality control of herbal materials and traditional medicines, and to facilitate characterization of novel metabolites are being developed. We hope these reports will spur further developments and enable natural products research to continue to expand chemical space for the benefit of humanity.

## References

[1] Gurusinghe S., Brooks T., Barrow R., Zhu X., Thotagamuwa A., Dennis P., Gupta V., Vanniasinkam T., Weston L. (2019). Technologies for the Selection, Culture and Metabolic Profiling of Unique Rhizosphere Microorganisms for Natural Product Discovery. Molecules.

[2] Juárez-Aragón M., Moreno-Ramírez Y., Guerra-Pérez A., Mora-Olivo A., Olazarán-Santibáñez F., Torres-Castillo J. (2019). Drying Effects on Phenolics and Free Radical-Scavenging Capacity of *Rhus pachyrrhachis* and *Rhus virens* Used in Traditional Medicine. Molecules.

[3] Molino A., Larocca V., Di Sanzo G., Martino M., Casella P., Marino T., Karatza D., Musmarra D. (2019). Extraction of Bioactive Compounds Using Supercritical Carbon Dioxide. Molecules.

[4] Mehariya S., Iovine A., Di Sanzo G., Larocca V., Martino M., Leone G., Casella P., Karatza D., Marino T., Musmarra D. (2019). Supercritical Fluid Extraction of Lutein from *Scenedesmus almeriensis*. Molecules.

[5] Yohannes A., Zhang B., Dong B., Yao S. (2019). Ultrasonic Extraction of Tropane Alkaloids from *Radix physochlainae* Using as Extractant an Ionic Liquid with Similar Structure. Molecules.

[6] Lee J., Ji S., Choi B., Choi D., Lee Y., Kim H., Kim G., Kim K., Lee Y., Baek N. (2018). UPLC-QTOF/MS-Based Metabolomics Applied for the Quality Evaluation of Four Processed *Panax ginseng* Products. Molecules.

[7] Yuan F., Cheng K., Gao J., Pan S. (2018). Characterization of Cultivar Differences of Blueberry Wines Using GC-QTOF-MS and Metabolic Profiling Methods. Molecules.

[8] Cabañas-García E., Areche C., Jáuregui-Rincón J., Cruz-Sosa F., Pérez-Molphe Balch E. (2019). Phytochemical Profiling of *Coryphantha macromeris* (Cactaceae) Growing in Greenhouse Conditions Using Ultra-High-Performance Liquid Chromatography–Tandem Mass Spectrometry. Molecules.

[9] Tsang M., Shaw P., Chu I., Cheng L., Wong E., Lau D., Lam C., Wong C. (2019). High-Throughput Immunological Analysis of Dictamni Cortex: Implication in the Quality Control of Herbal Medicine. Molecules.

[10] Pearce A., Copp B., Molinski T. (2019). Enantiomeric Variability of Distaminolyne A. Refinement of ECD and NMR Methods for Determining Optical Purity of 1-Amino-2-Alkanols. Molecules.

